# Decrease in Circulating Fatty Acids Is Associated with Islet Dysfunction in Chronically Sleep-Restricted Rats

**DOI:** 10.3390/ijms17122102

**Published:** 2016-12-14

**Authors:** Shanshan Zhan, Yangyang Wu, Peng Sun, Haiyan Lin, Yunxia Zhu, Xiao Han

**Affiliations:** Key Laboratory of Human Functional Genomics of Jiangsu Province, Jiangsu Diabetes Center, Nanjing Medical University, Nanjing 210029, China; zsskr@sina.com (S.Z.); yynjmu@gmail.com (Y.W.); sunpeng@njmu.edu.cn (P.S.); linda@njmu.edu.cn (H.L.); zhuyx@njmu.edu.cn (Y.Z.)

**Keywords:** sleep-restriction, islet function, circulating fatty acids, stress response, metabonomics

## Abstract

Previous studies have shown that sleep restriction-induced environmental stress is associated with abnormal metabolism, but the underlying mechanism is poorly understood. In the current study, we investigated the possible lipid and glucose metabolism patterns in chronically sleep-restricted rat. Without changes in food intake, body weight was decreased and energy expenditure was increased in sleep-restricted rats. The effects of chronic sleep disturbance on metabolites in serum were examined using ^1^H NMR metabolomics and GC-FID/MS analysis. Six metabolites (lipoproteins, triglycerides, isoleucine, valine, choline, and phosphorylcholine) exhibited significant alteration, and all the fatty acid components were decreased, which suggested fatty acid metabolism was impaired after sleep loss. Moreover, increased blood glucose, reduced serum insulin, decreased glucose tolerance, and impaired glucose-stimulated insulin secretion of islets were also observed in sleep-restricted rats. The islet function of insulin secretion could be partially restored by increasing dietary fat to sleep-disturbed rats suggested that a reduction in circulating fatty acids was related to islet dysfunction under sleep deficiency-induced environmental stress. This study provides a new perspective on the relationship between insufficient sleep and lipid/glucose metabolism, which offers insights into the role of stressful challenges in a healthy lifestyle.

## 1. Introduction

Since an increasing number of people extend their work well into the night and curtail or delay sleep in order to meet the demands of a rapidly developing global economy, circadian rhythm misalignment has become a social problem nowadays [1]. A line of studies has demonstrated that sleep loss is tightly coupled with an increased risk of cardiovascular disease [2], inflammation [3], neuroendocrine dysfunction [4], metabolic syndrome [5] and type 2 diabetes [6]. Type 2 diabetes is typically characterized by absolute or relative insulin deficiency [7]. Proper insulin secretion, which rises during the day and falls during the night, is regulated by the circadian rhythm system [8]. The negative effects of insufficient sleep on insulin secretion have already received considerable attention. It was reported that short sleepers exhibit impaired glucose tolerance, decreased insulin sensitivity, and hyperglycemia, which suggests that they suffer from an insulin secretory deficiency [9]. However, as it is well-known that pancreatic β cell dysfunction results in abnormal insulin secretion, less studies have described islet β cell function directly under sleep debt.

In this present study, the platform in water model, which was a classical animal model [10], was used to imitate chronic sleep restriction (CSR) in rats. We aimed to assess the effects of sleep restriction and environmental stress on metabolism and islet function, thereby exploring the underlying mechanism of this relationship.

## 2. Results

### 2.1. Metabolic Features of CSR Rat Model

**Figure 1:**
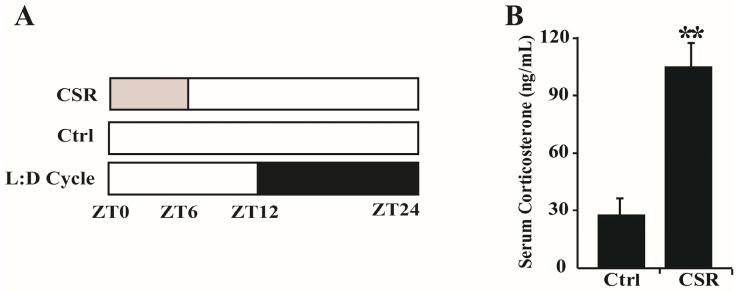
A rat model of CSR was successfully constructed: (**A**) Schematic overview of sleep restriction protocol for CSR and control rats. For CSR rats, period of forced wakefulness was shown in light grey from ZT0 to ZT6 each day while control rats were left undisturbed; (**B**) Serum concentration of corticosterone was measured after CSR., ** *p* < 0.01 vs. control. *n* = 10.

After four weeks of sleep restriction, all the rats survived, no one became moribund, had to be euthanized or substituted by additional animals. Serum corticosterone level was measured firstly because it is considered a biomarker of sleep loss [11]. Indeed, the concentration of serum corticosterone (Figure 1B) was significantly increased in CSR rats compared to controls. This suggested that the chronic-sleep-restriction model was successfully constructed.

**Figure 2:**
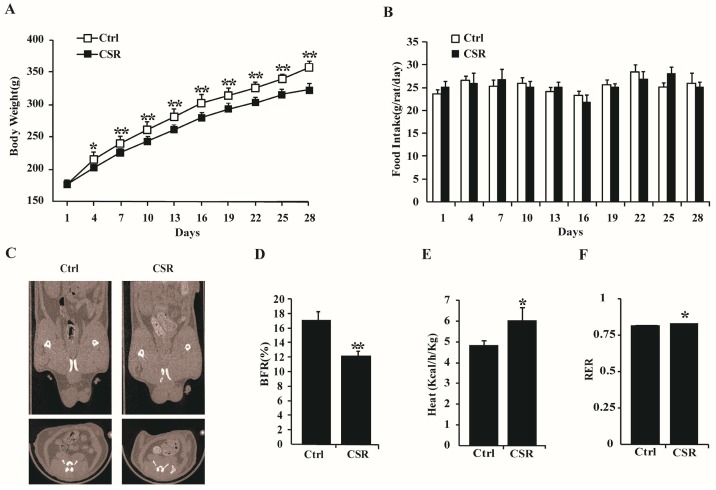
Chronic sleep restriction increased basal metabolism: Body weight (**A**); and food intake (**B**) of control and CSR rats were monitored during chronic sleep loss. White squares = control group; black squares = CSR group. *n* = 10; (**C**) Reconstructive micro-CT scanning images of abdominal fat in control and CSR rats. *n* = 5; (**D**) BFR was calculated from (**C**). Heat production (**E**); and RER (**F**) were measured after CSR by metabolic cages. * *p* < 0.05, ** *p* < 0.01 vs. control. *n* = 5.

Body weight gain was reduced in CSR rats (Figure 2A), although the groups did not exhibit obvious differences regarding food intake (Figure 2B). Further, the decreased fat mass in CSR rats was evident in the reconstructed micro-CT-scan images intuitively (Figure 2C), and the body fat radio (BFR) was 30% lower in CSR rats than in controls by quantitative calculation (Figure 2D). Reduced fat mass unaccompanied by changes in food intake indicated higher energy expenditure in CSR rats than in controls. Indeed, sleep restriction significantly increased heat production (Figure 2E) and respiratory exchange ratio (RER) in rats (Figure 2F).

### 2.2. ^1^H NMR Metabolomics and GC-FID/MS Analysis

**Figure 3:**
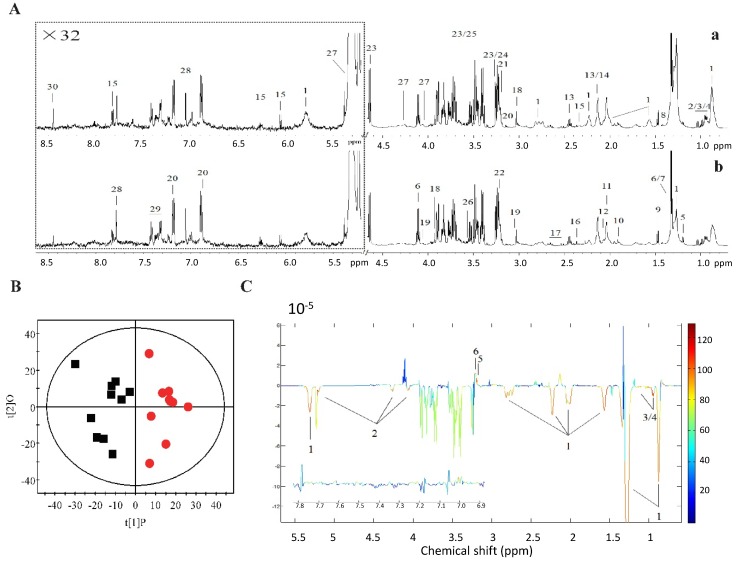

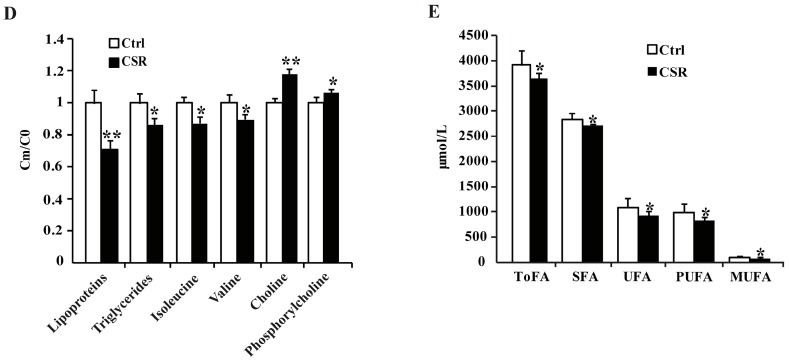
Metabolomic analysis of chronic sleep restriction: (**A**) Typical ^1^H NMR spectra (600 MHz) obtained from serum of control (**a**) and CSR rats (**b**) after sleep deficiency. The keys for the metabolites were given in Table S1. Cross-validated OPLS-DA scores plots (**B**); and the corresponding loadings plots (**C**) from ^1^H NMR spectra of control and CSR rats. Black squares = control group; red dots = CSR group. (OPLS-DA: *Q*^2^ = 0.493; CV-ANOVA: *p* = 0.029). Keys: 1, lipoproteins; 2, isoleucine; 3, valine; 4, choline; 5, phosphorycholine; 6, triglycerides; (**D**) The relative quantitative ratios of significantly changed metabolites in (**C**); (**E**) Fatty acid compositions obtained from GC-FID/MS analysis of serum. ToFA: total fatty acids; SFAs: saturated fatty acids; UFAs: unsaturated fatty acids; MUFAs: monounsaturated fatty acids; PUFAs: polyunsaturated fatty acids. White squares = control group; black squares = CSR group. * *p* < 0.05, ** *p* < 0.01 vs. control. *n* = 10.

**Figure 4:**
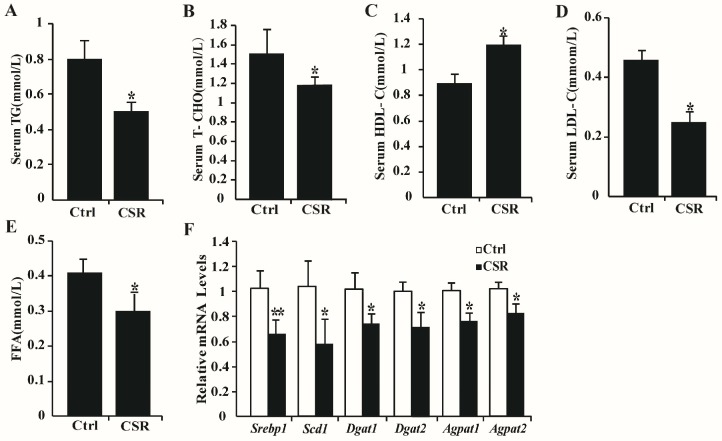
Chronic sleep restriction decreased serum lipid metabolic parameters and expression of lipid metabolic genes in the liver: Serum concentration of: Tg (**A**); T-CHO (**B**); HDL-C (**C**); LDL-C (**D**); and FFAs (**E**) were measured after CSR. (**F**) Relative mRNA levels of lipid metabolic genes in the liver of control and CSR rats. White squares = control group; black squares = CSR group. * *p* < 0.05, ** *p* < 0.01 vs. control. *n* = 5.

Since CSR caused energy imbalance in rats CSR, the broad effect of short sleep on serum metabolites was explored. Serum samples from control and CSR rats were collected and analyzed using ^1^H NMR metabolomics. Thirty different metabolites were assigned in the ^1^H NMR spectra of serum (Figure 3A, Table S1), which contained signals from lipoproteins, glycoproteins, glucose, amino acids, and choline metabolites. OPLS-DA was carried out for ^1^H NMR spectral data sets from serum (Figure 3B). The model quality (*Q*^2^ = 0.493, *p* = 0.029) indicated a distinct difference between metabonomic profiles in control and CSR rats. Six of the detectable metabolites were changed significantly between two groups, including lipoproteins, triglycerides, isoleucine, valine, choline, and phosphorylcholine (Figure 3C, Table S2). Remarkable depletions in the levels of lipoproteins, triglycerides, isoleucine, valine and marked elevations in the levels of choline and phosphorylcholine were observed in serum of CSR rats compared with control ones (Figure 3D). These data suggested that the key changes between control and CSR rats in metabolism might be fatty acid metabolism. The fatty acid compositions in serum were further investigated using GC-FID/MS analysis, which showed a significant decline of all fatty acids in CSR rats, including ToFAs, SFAs, MUFAs, and PUFAs (Figure 3E, Table S3). Among the changed fatty acid components, myristic acid (C14: 0), palmitic acid (C16: 0), palmitic acid (C16: 1), stearic acid (C18: 0), oleic acid (C18: 1), linoleic acid (C18: 2), α-linolenic acid (C18: 3), eicosapentaenoic acid (C22: 5), and docosahexaenoic acid (C22: 6) were significantly reduced in CSR rats. In accordance with the results shown above, serum levels of triglyceride (Tg), total-cholesterol (T-CHO), low density lipoprotein-cholesterol (LDL-C), and free fatty acids (FFAs) were markedly decreased, while high density lipoprotein-cholesterol (HDL-C) was markedly increased in CSR rats (Figure 4A–E). The mRNA levels of lipid metabolic genes were examined in the liver. The expression of *Srebp1*, *Scd1*, *Dgat1/2*, and *Agpat1/2* were significantly lower in CSR rats than in controls (Figure 4F), indicating decreased lipid biosynthesis after sleep loss. No marked changes were observed in expression levels of genes involved in glucose metabolism (Figure S1). Hence, these results highlighted the broad effect of chronic sleep restriction on lipid homeostasis.

### 2.3. Dysfunction of CSR Rat Islets

**Figure 5:**
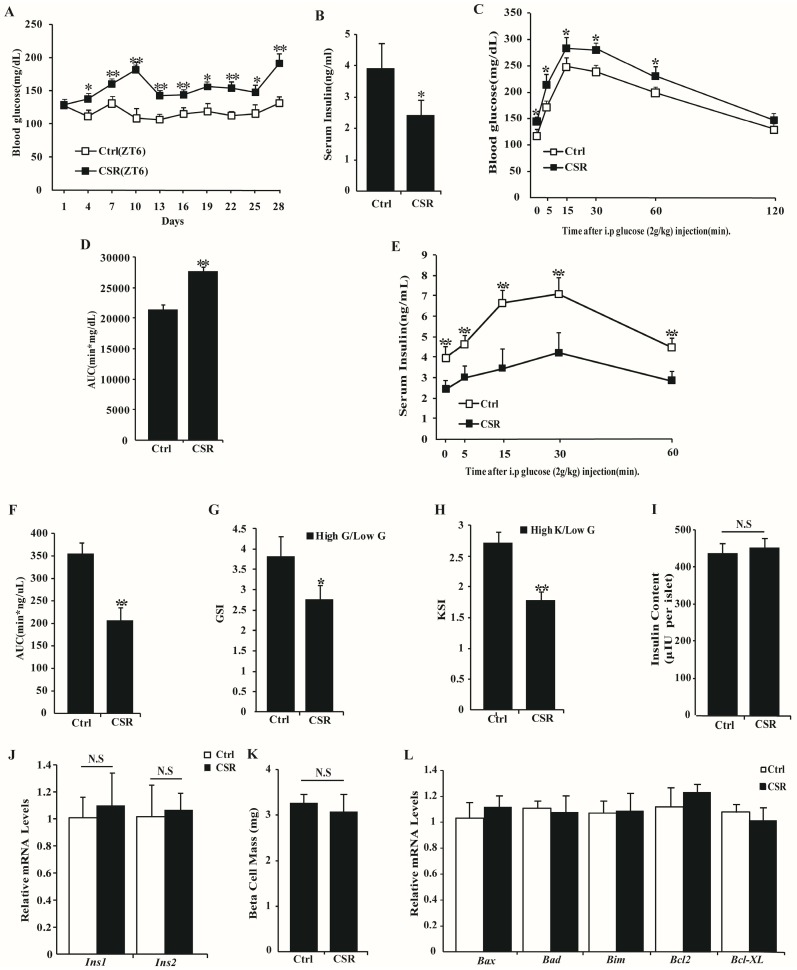
Chronic sleep restriction impaired islet function: (**A**) Fasting blood glucose of control and CSR rats were monitored during chronic sleep loss (*n* = 10); (**B**) Fasting serum insulin of control and CSR rats were measured after chronic sleep disturbance (*n* = 10); (**C**) IPGTTs were performed at the end of CSR treatment (i.p = intraperitoneal, *n* = 5); (**D**) The AUC of (**C**); (**E**) Analysis of serum insulin levels during IPGTTs (*n* = 5); (**F**) The AUC of (**E**). Isolated islets from control and CSR rats were analyzed for insulin secretion by incubation in response to stimulation with: 3.3 mM glucose (Low **G**) (**G**); and 16.7 mM glucose (High **G**) or 50 mM KCL (High **K**) (**H**) (*n* = 5); (**I**) Analysis of insulin content in isolated islets used in (**G**); (**J**) Relative mRNA levels of *Ins1* and *Ins2* in islets of control and CSR rats (*n* = 5); (**K**) The mean β cell mass in islets (*n* = 5); (**L**) Relative mRNA levels of genes involved in apoptosis pathways in islets (*n* = 5). White squares = control group; black squares = CSR group. * *p* < 0.05, ** *p* < 0.01 vs. control. N.S = no significant change.

At the same time, fasting blood glucose level was significantly increased (Figure 5A) and serum insulin level was markedly reduced (Figure 5B) in rats after sleep disturbance. CSR rats exhibited significantly higher blood glucose levels and lower insulin concentration than controls during IPGTTs (Figure 5C–F). A glucose-stimulated insulin secretion (GSIS) assay revealed a marked decrease in insulin secretion in isolated islets of CSR rats under high glucose treatment compared to controls (Figure 5G). In addition, pancreatic β cell function was also assessed using direct depolarization with KCL. Marked impairment in insulin secretion was found in islets of CSR rats for KCL-induced insulin secretion (KSIS) assay after being normalized with insulin content (Figure 5H). Insulin content (Figure 5I) and relative mRNA levels of the *Ins1* and *Ins2* genes (Figure 5J) were not significantly changed. Also, β cell mass (Figure 5K) and relative mRNA levels of *Bax*, *Bad*, *Bim*, *Bcl2* and *Bcl-xl* genes (Figure 5L) were not markedly different between the two groups. These data indicated that despite insulin synthesis and islets turnover, insulin secretion was decreased in CSR rats.

**Figure 6:**
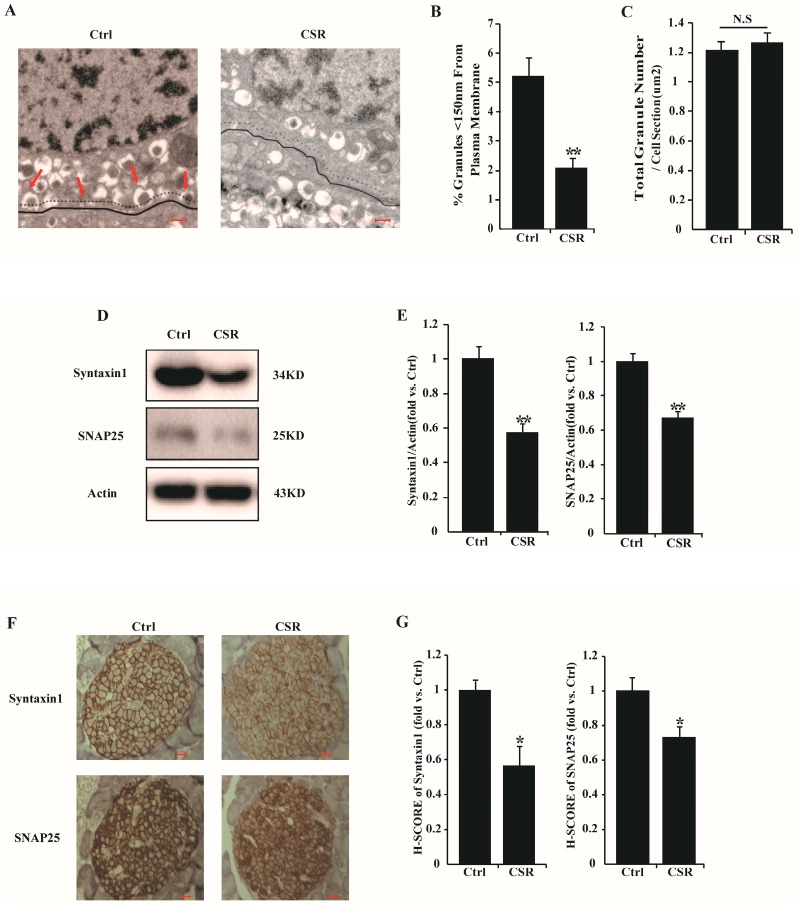
Chronic sleep restriction reduced insulin granules docking at pancreatic β cell membrane: (**A**) Representative electron micrographs of control and CSR rats pancreatic β cells were shown. Pancreatic β cell membrane and 150 nm distance from plasma membrane were traced in black solid line and dashed line, respectively. Arrowheads point to examples of docked granules, scale bar = 300 nm.; (**B**) The percentage of insulin granules localized to the plasma membrane (<150 nm) within β cells; (**C**) Average total insulin granule number was calculated, normalized to cell section; (**D**) Protein levels of Syntaxin1 and SNAP25 in islets; (**E**) Quantification of (**D**); (**F**) IHC staining of Syntaxin1 and SNAP25 in islets, scale bar = 10 µm; (**G**) Quantification of (**F**). * *p* < 0.05, ** *p* < 0.01 vs. control. N.S = no significant change. *n* = 5.

The primary common process in GSIS and KSIS is insulin granule exocytosis. The last steps in this process are controlled by correct docking and fusion of insulin granules to the plasma membrane of pancreatic β cells. GSIS and KSIS were both decreased in CSR rats, which indicated impaired insulin granule activity. To investigate our hypothesis, electron microscopic analysis of β cells was performed in islets isolated from control and CSR rats. Sleep loss reduced insulin granule docking (Figure 6A), and it resulted in 60% fewer docking granules in close proximity (<150 nm) to the plasma membrane of β cells in CSR rats compared to controls (Figure 6B), whereas the number of mature insulin granules was not significantly different between the two groups (Figure 6C). Soluble *N*-ethylmaleimide-sensitive factor attachment protein receptor (SNARE) protein complex is responsible for the fusion step of insulin granule activity [12]. It was found that protein expression (Figure 6D,E) and staining levels (Figure 6F,G) of Syntaxin1 and SNAP25, two important members of SNARE, were both decreased in islets of CSR rats. Thus, islet dysfunction was caused by reduced insulin granule docking in CSR rats.

### 2.4. Increased Dietary Fat Prevented Islets Function of CSR Rats

**Figure 7:**
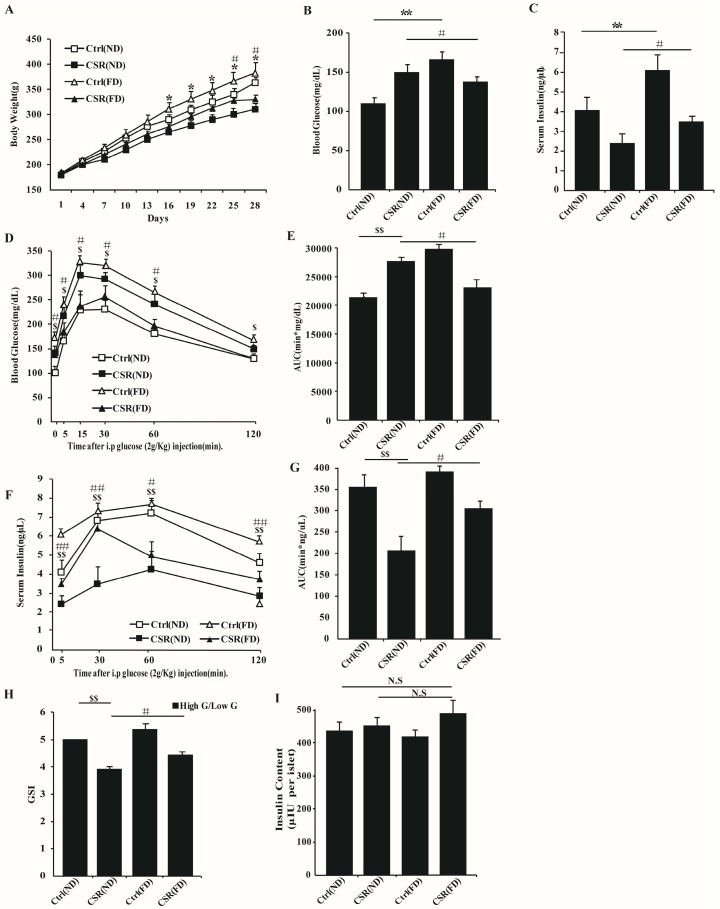
Increased dietary fat restored islet dysfunction induced by chronic sleep restriction partially: (**A**) Body weight was monitored during CSR in ND and FD groups (*n* = 10). Fasting blood glucose (**B**); and serum insulin (**C**) were measured after CSR in ND and FD groups (*n* = 10); (**D**) IPGTTs were performed at the end of CSR treatment (*n* = 5); (**E**) The AUC of (**D**); (**F**) Analysis of serum insulin levels during IPGTTs (*n* = 5); (**G**) The AUC of (**F**); (**H**) Isolated islets were analyzed for insulin secretion by incubation in response to stimulation with 3.3 mM glucose (Low **G**) and 16.7 mM glucose (High **G**) (*n* = 5); (**I**) Analysis of insulin content in isolated islets used in (**H**) (*n* = 5). ND = Normal Diet; FD = diet supplemented with fatty acids. White squares = Ctrl(ND) group; black squares = CSR(ND) group; white triangle = Ctrl(FD) group; black triangle = CSR(FD) group. * *p* < 0.05, ** *p* < 0.01, Ctrl(FD) vs. Ctrl(ND) rats ; ^$^ *p* < 0.05, ^$$^ *p* < 0.01, Ctrl(ND) vs. CSR(ND) rats; ^#^ *p* < 0.05, ^##^ *p* < 0.01, CSR(FD) vs. CSR(ND) rats. N.S = no significant change.

Therefore, circulating fatty acids was decreased and islets function was impaired in sleep-restricted rats. GPR40, an FFAs receptor on the islet membrane, promotes FFAs-induced insulin secretion [13]. The expression level of GPR40 was reduced in the islets of CSR rats compared to controls (Figure S4A). We assumed that reduced insulin release induced by insufficient sleep is due to the decreased level of serum FFAs. In order to elevate the concentration of circulating FFAs, a diet supplemented with fatty acids (FD) was substituted for the normal standard diet (ND) to feed chronically sleep-restricted rats. Interestingly, compared with CSR(ND) rats, CSR(FD) rats had increased body weight (Figure 7A), lower levels of blood glucose (Figure 7B), and higher levels of serum insulin (Figure 7C). The IPGTT assay showed that glucose tolerance was improved (Figure 7D,E) and levels of serum insulin during IPGTTs were elevated in CSR(FD) rats (Figure 7F,G). Furthermore, islet GSIS function was partially restored in CSR(FD) rats compared to CSR(ND) rats (Figure 7H), whereas the two groups did not exhibit a significant difference with insulin content (Figure 7I).

## 3. Discussion

Although it is clear that sleep loss is associated with body weight changes, the nature of this relationship is still under debate. Several reports have demonstrated that sleep deficiency induces obesity [14,15], whereas others have found that it attenuates weight gain [16,17]. Different models might contribute to these controversial results. In some models, like what we used in the present study, animals endured not only sleep restriction but also stress response.

Stress can be considered as a reaction between brain and body to response environmental or internal stimulation [18]. It is undeniable that sleep/EEG data is the gold standard to assess sleep/awake status, and sleep/EEG datawas shown in many studies about sleep. However, in our study, we considered sleep restriction as a model that could induce stress and discussed metabolic changes under stress induced by sleep debt. As a central nervous excitation enhancer, serum corticosterone is an important hormone responsible for stress in rat. Circadian rhythms and sleep play key roles in regulating serum corticosterone concentration. Increased serum corticosterone is considered a biological indicator of sleep restriction [11]. Compared to control rats, higher concentration of serum corticosterone confirmed that sleep restriction indeed caused stress in rats. Stressful situation causes body weight loss [19,20]. As a stressor, chronic sleep restriction reduced body weight and increased energy expenditure in rats despite a level of food intake equivalent to that of controls in our study, which were similar to other previous studies [21,22]. The attenuation of body weight gain appears to be a direct consequence of increased energy expenditure. Being awake induces more energy consumption and higher sympathetic autonomic nervous system activity than being asleep [23,24].

As decreased body weight was found in CSR rats, we further investigated the metabolomics, the profiling of small-molecule metabolites to directly distinguish metabolic phenotypes that are associated with environmental effects. Previous metabolomics-based researched on human beings and rats have confirmed that a series of metabolites can be altered by sleep restriction [25,26]. Plasma lipids bind with apolipoproteins to form lipoproteins which participate in lipid transport and metabolism. Long-term sleep restriction may affect blood lipoprotein levels [27]. We found that the amount of serum lipoproteins was reduced in CSR rats. Chronic sleep loss may decrease triglyceride levels by suppressing *Srebp1* and *Scd1* expression in the liver. Increased cholesterol absorption and utilization may inhibit *Srebp1* transcription and ultimately reduce lipid biosynthesis [28]. In this study, decreased levels of lipoprotein and triglyceride were consistent with body weight loss in CSR rats. A study found that isoleucine and valine were 20% and 14% lower in lean compared to obese people, respectively [29]. Likewise, reduced levels of isoleucine and valine were observed in CSR rats. Moreover, sleep loss had catabolic effects on whole body protein to support energy needs [30]. Choline is an important precursor of phosphorycholine and has an affinity for lipids. To prevent the abnormal accumulation of lipids in the liver, it can promote lipid removal from the liver or improve the utilization of fatty acids in the liver. Choline can also prevent the deposition of cholesterol in blood vessels in order to improve the absorption and utilization of lipids. Choline and phosphorylcholine were decreased after long-term sleep debt, which indicated a higher rate of fatty acid clearance in CSR rats.

It is noteworthy that we did not find significantly changed metabolites in glycolysis and gluconeogenesis. This is consistent with a previous study [31] and further suggested that amino acids and lipids played key role under sleep restriction. Hence, the metabolomic analysis indicated that chronic sleep curtailment has a prominent effect on fatty acid metabolism. Indeed, we pointed out all the fatty acid compositions were decreased in CSR rats. *Scd1* accelerates the biosynthesis of MUFAs, thereby increasing triglyceride synthesis, and results in an elevated level of serum triglyceride. In the present study, *Scd1* expression was reduced in liver of CSR rats, which in turn reduced MUFAs and triglyceride synthesis. Decreased levels of UFAs and PUFAs in the serum unaccompanied by a significant difference in food intake between the groups indicated an increased uptake of UFAs and PUFAs after sleep loss. Increased UFAs and PUFAs absorption can inhibit the transcription of *Srebp1* and de novo lipid synthesis. C18: 2 and C18: 3 can generate higher carbon chain PUFAs by a series of enzymatic reactions and can accelerate adipogenesis. In our study, stress weakened these effects. The reduced lipid levels suggested that lipolysis and fatty acid oxidation were important bioenergetic pathways in CSR rats. Recent studies have reported that sleep restriction induced stress modulates lipid homeostasis [16,31,32], and our results enhanced the understanding of the effect of chronic sleep disturbance on fatty acid composition.

As nutritive and signal factors, fatty acids are important to maintaining normal insulin release [33,34]. Low circulating FFAs levels in rats significantly impairs GSIS function. Insulin secretion of islets is decreased by administering nicotinic acids in order to deplete FFAs [35,36]. GPR40 is considered a special membrane receptor of FFAs in islets and plays a key role in the fatty acidmediated augmentation of insulin secretion. The activation of GPR40 in pancreatic β cells potentiates GSIS [13]. The expression of GPR40 in islets of T2D patients is lower than normal people [37]. The GC-FID/MS analysis showed that all the detectable fatty acids were medium- or long-chain fatty acids (C14-C22), so they could be ligands of GPR40. Abnormal lipid metabolism under sleep curtailment was further demonstrated by reduced concentration of circulating FFAs accompanied by low protein expression level of GPR40 in islets.

It was found that glucose metabolism was impaired in CSR rats. The absence of obvious differences between control and CSR rats during IPITTs (Figure S2) suggested that chronic sleep loss caused pancreatic islet dysfunction rather than insulin sensitivity. Pancreatic islets of CSR and control rats exhibited no significant differences in morphology (Figure S3A). Both the GSIS and KSIS functions of pancreatic β cells were markedly impaired in CSR rats. KSIS bypasses steps involving energy metabolism in GSIS. AMPK and mTOR are key regulators of energy homeostasis. Surprisingly, elevated expression levels of p-AMPK together with decreased expression levels of p-mTOR in islets of CSR rats following exposure to high glucose (Figure S3B) showed an increase in catabolism and a reduction in anabolism. Simultaneously, whole-cell ATP content and OCR (Figure S3C,D) were increased in CSR islets under high glucose condition. These data indicated the presence of a high energy expenditure state in islets following sleep loss. Previous studies have reported that sleep restriction increased EEG delta waves in non-rapid eye movement by activating the AMPK signaling pathway in hypothalamus [38] and that sleep loss increased neural activity and energy consumption in the brain [39,40]. Our results provided a new basis for energy metabolism promoted by stress under restricted sleep in peripheral cells. The evidences outlined above may seem inconsistent with impaired GSIS; however, the higher energy expenditure of islets under sleep loss may be a compensatory effect responsible to stress. Thus, islet dysfunction induced by restricted sleep may be due to impaired insulin granule exocytosis rather than impaired energy metabolism.

The priming, transportation, docking, and fusion of insulin granules to the plasma membrane are governed by the SNARE complex [12,41]. The pancreatic β cells of CSR rats exhibited a severely impaired docking of insulin granules and a lack of alteration in total insulin granule mass, which was consistent with the unchanged insulin content. Syntaxin1 and SNAP25 are directly involved in insulin granule exocytosis [42]. Decreased expression levels of Syntaxin1 and SNAP25 in islets of GK rats and obese Zucker rats have been proposed to reflect a reduction in insulin secretion [43,44]. We believe the markedly reduced protein expression levels of Syntaxin1 and SNAP25 in islets of CSR rats are responsible for the low insulin secretion after sleep debt. Taken together, decreased insulin granule docking and exocytosis could be strongly associated with impaired islet function in CSR rat model.

Nutritional status could be an important part of stress [45,46]. As decreased circulating FFAs and GPR40 expression in islets could contribute to islets dysfunction in CSR rats, we further explored whether FFAs supplements could reverse sleep restriction-induced dysfunction of islets. We used FD as an energy resource to complement circulating FFAs during sleep loss. Interestingly, after 4-week treatment of FD, glucose tolerance was improved and islet GSIS function was partially recovered in CSR (FD) rats compared to CSR (ND) rats. It was confirmed that circulating FFAs was significantly increased (Figure S4B), and the expression of GPR40 was also elevated (Figure S4C) in islets of CSR (FD) rats. These results indicated that increased levels of circulating FFAs contributed to the recovery of islet function in CSR rats. Horohov et al showed that feeding sleep-restricted rats a diet enriched in fatty acids prevented sleep restrictioninduced immune suppression [47], which also confirmed the beneficial role of FFAs nutrition in inadequate sleep. Thus, it could be drawn that appropriated fatty acids supplement might provide benefit for glucose metabolism in sleep-restricted individuals.

As an exogenous stressor, chronic sleep curtailment has become a common problem and has been shown to be associated with type 2 diabetes. Our study used chronic sleep restriction model to simulate human’s daily life in modern society and showed that chronic sleep loss changed fatty acid metabolism and impaired islet function of insulin secretion in rats. The effect of chronic sleep restriction on islet dysfunction is based on the decreased circulating FFAs and reduced docking of insulin granules. While increasing dietary fat, the insulin secretion of CSR rats islets was partially restored. To our knowledge, this is the first report to reveal the mechanism underlying sleep lossinduced islet dysfunction and to highlight the importance of circulating FFAs. Although we acquired these data in animal experiment and further clinical investigations are needed, we believed that this conclusion would be relevant to humans, and our study may provide new insights into the role of sufficient sleep and stress response in a healthy lifestyle.

## 4. Materials and Methods

### 4.1. Animals

In order to control and eliminate interferences of mating, development and physiological cycle in females [48,49], all experiments were performed with male Sprague-Dawley rats (150 ± 10 g) obtained from Sino-British SIPPR/BK Lab Animal Ltd. (Shanghai, China) and housed under temperature-, humidity-, and light-controlled conditions (22 °C, 50% humidity, 12:12 h light-dark cycle, lights on at 08:00). All the rats were caged individually, one rat in one cage. Food and water were provided ad libitum. After acclimatization for one week, rats were divided into two groups: control (*n* = 10) and chronic sleep restriction (CSR, *n* = 10) groups. The high-fat diet was purchased from Research Diets (D12492, New Brunswick, NJ, USA). Procedures involving animals and their care in this study were conducted inconformity with NIH guidelines (NIH Pub. No. 85-23, revised 1996) and was approved by Animal Care Committee of Nanjing Medical University, China (20130901, 1 Sepember 2013).

### 4.2. Sleep Restriction

Chronic sleep restriction was induced by using the platform in water model as previously described [16]. Rat in the CSR group was placed on a circular platform (4.5 cm in diameter, 5 cm in height), which could not rotate, in a metallic cage (23 cm × 23 cm × 35 cm) filled with water. The platform was 1 cm above the water. Muscular atonia caused rat to fall into the water and awaken. Control rat was placed in a similar cage containing a circular platform (15 cm in diameter, 5 cm in height) without water in it and was maintained in the same experimental environment as CSR rat. The water in the cages was changed daily. All rats were habituated to the experimental conditions by placing them in the corresponding cages for 1 h on three consecutive days before the experiment. The rats from both groups were kept in the metallic cages for 6 h per day (08:00–14:00) four weeks. All the rats were fasted during this period, after which they behaved freely (Figure 1A).

### 4.3. Measurement of Metabolic Factors

Body weight was measured every two days. Food intake was measured by subtracting the weight of the feed cup from the previous days’ weight. Blood glucose levels were measured using a glucometer (Abbot, Alameda, CA, USA). Heat production and respiratory exchange ratio were measured using metabolic cages. Serum samples were collected immediately after sleep restriction within one hour, when Serum corticosterone concentration was between 14 and 15 p.m. was determined with ELISA kits (Xinqidi Biological Technology, Wuhan, China). Circulating total cholesterol, triglycerides, HDL cholesterol, and free fatty acids were assessed with biochemical kits (Nanjing Jiancheng Bioengineering Institute, Nanjing, China).

### 4.4. CT Measurements

The abdominal fat of rats was measured using a micro-computed tomography scanner (SkyScan1176-X-ray, Brucker, Germany). Rats were anesthetized with isoflurane and scanned from the tailbone to the lungs. Fat images and body fat ratio were quantified semiautomatically on dedicated software (NRecon, Kontich, Belgium).

### 4.5. ^1^H NMR Spectroscopy and Fatty Acid Analysis

Blood samples of all rats were collected from the orbital venous plexus and centrifuged at 3000 rpm for 10 min at 4 °C to obtain serum. All the serum samples were immersed in liquid nitrogen immediately after collection and stored at −80 °C until NMR acquisition was performed. ^1^H NMR spectra were recorded, NMR data were analyzed, and the composition of fatty acids in serum was examined as previously described [50]. See more details in Supplementary Materials.

### 4.6. Glucose Tolerance Test and Insulin Tolerance Test

All rats were fasted overnight before undergoing an intraperitoneal glucose tolerance test (IPGTT) or intraperitoneal insulin tolerance test (IPITT). Fasting blood glucose levels of all rats from the tail vein were measured as initial blood glucose. For IPGTT and IPITT, rats received an intraperitoneal injection of glucose (2 g/kg) or insulin (1 U/kg), and blood samples were taken at 5, 15, 30, 60, 120, and 180 min (IPITT only). In addition, blood samples were collected during IPGTT for insulin measurements. Insulin levels were measured using rat insulin ELISA kits (Millipore, Billerica, MA, USA).

### 4.7. Islet Isolation and Insulin Secretion Assay

Pancreatic islets were isolated from CSR and control rats by collagenase XI (Sigma-Aldrich, St. Louis, MO, USA) digestion and histopaque (Sigma-Aldrich) separation from acinar and ductal tissue. Islets were then hand-picked and cultured for 8 h in RPMI 1640 plus 10% fetal bovine serum at 37 °C and 5% CO_2_ before the insulin secretion assay. Insulin secretion studies were performed in overnight cultured islets and were analyzed in six repeated samples with seven size-matched islets each. Islets were washed and preincubated in Krebs-Ringer bicarbonate HEPES buffer (KRBH) with 3.3 mM glucose for 1 h, followed by 1 h of incubation with KRBH buffer containing 16.7 mM glucose or 50 mM KCL, respectively. Secreted insulin levels and total insulin content were analyzed using an RIA method with insulin detection kits (Beijing North Institute of Biological Technology, Beijing, China).

### 4.8. Western Blotting

Isolated islets from CSR and control rats were cultured overnight. Five rats per group were selected and 500 islets per rat were extracted randomly for protein. Protein content determination and western blot analyses were performed as previously described [51]. Immunoblots were probed with the following antibodies purchased from Santa Cruz Biotechnology (Santa Cruz, CA, USA): mouse anti-Syntaxin1 diluted 1:1000, mouse anti-SNAP25 diluted 1:1000 and rabbit anti-GPR40 diluted 1:1000. They were quantified relative to levels of control protein, mouse anti-β-actin diluted 1:3000 (Sigma-Aldrich).

### 4.9. RNA Isolation, RT-PCR, Real-Time Quantitative PCR

Isolated islets of CSR and control rats were cultured overnight, and 200 islets per rat were extracted for total RNA. For mRNA determination, oligo-dT was used as the reverse primer, and first-strand cDNA synthesis was performed using 1 μg total RNA (Promega, Madison, WI, USA). Quantitative RT-PCR was performed using the SYBR Green PCR Master Mix and LighteCycler480 II Sequence Detection System (Roche, Basel, Switzerland). All detections on mRNA expression were normalized by tissue weights and internal control gene β-actin. Sequences of the primers (5′–3′):

Srebp1: F: CGCTACCGTTCCTCTATCA
R: CTCCTCCACTGCCACAAG
Scd1: F: GCTAATATCTGGGTGTAATC
R: GGCTGGGTCATAGTTGTA
Agpat1: F: CGACCTGCTTGGAATGAT
R: AGCGTCTCCTGTGCGTTT
Agpat2: F: GCTGCTGTTGCTGCTTGT
R: CGTCCACCTCCAGTTTCTT
Dgat1: F: AGTGGGTTCCCTGTTTGC
R: TCTCGGTAGGTCAGGTTGT
Dgat2: F: GGAGATTGGCATCGTGAA
R: CAGGTGAGGCTTGGTTGG
Ins1: F: CATAGACCATCAGCAAGCAGG
R: GAAGAAACCACGTTCCCCAC
Ins2: F: TGTCAAACAGCACCTTTGTGG
R: GTGCCAAGGTCTGAAGGTCAC
Bax: F: GGCGATGAACTGGACAAC
R: TCCCGAAGTAGGAAAGGAG
Bad: F: GAGGAAGATGAAGGGATGG
R: GGACTCGCAACTTAGCACA
Bim: F: CCTACAGACAGAATCGCAAGA
R: GACGGAAGATGAATCGTAACAG
Bcl2: F: CGGGAGAACAGGGTATGA
R: CAGGCTGGAAGGAGAAGAT
Bcl-xl: F: GGTATTGGTGAGTCGGATTG
R: TGGACGGTCAGTGTCTGG
G6pase: F: TCCACCTTGACACTACACCC
R: GGGACGGTCGCACTCTT
Pck1: F: TGTTGGCTGGCTCTCACTG
R: ACCTTTGGGGATGGGCAC
Pepck: F: AGTCACCATCACTTCCTGGAAGA
R: GGTGCAGAATCGCGAGTTG
Glut2: F: ACACCAGCACATACGACA
R: CAAAGAACGAGGCGACTA
β-actin: F: CCATGTTCCAAAACCATTCC
R: GGGCAACCTTCCCAATAAAT

### 4.10. Oxygen Consumption Measurements

The oxygen consumption rate (OCR) of islet suspensions was determined by polarography using Clark oxygen electrodes (Strathkelvin Instruments, North Lanarkshire, UK) as previously described [52].

### 4.11. ATP Measurements

ATP levels of isolated islets were measured using a commercially available ATP determination kit according to the manufacturer’s instructions (Beyotime Institute of Biotechnology, Shanghai, China).

### 4.12. Transmission Electron Microscopy

Isolated islets from CSR and control rats (*n* = 5 per group) were fixed in 4% paraformaldehyde, postfixed in 1% osmium tetroxide, dehydrated through a series of increasing alcohol concentrations, and embedded in epoxy resin. Ultrathin sections were stained with uranyl acetate citrate and examined with a transmission electron microscope (JEOL Ltd., Tokyo, Japan).

### 4.13. Morphometric and Histologic Analysis

Histologic studies were conducted using pancreas samples from CSR and control rats (*n* = 6 per group). The samples were fixed overnight in 4% paraformaldehyde and then processed for paraffin embedding, sectioning, and H&E staining. Tissue sections were deparaffinized in xylene and rehydrated in ethanol. Sections were processed using the avidin-biotin-peroxidase method with anti-Syntaxin1 (sc-12736) and anti-SNAP25 (sc-20038) antibodies. Microphotographs were captured with an Olympus microscope. The β cell mass for each rat was measured by first obtaining the fraction of the cross-sectional area of pancreatic tissue positive for insulin staining and then multiplying this by the pancreatic weight.

### 4.14. Statistical Analysis

Results are presented as averages and S.E. The statistical significance of differences between groups was determined using Student’s *t*-test or, where appropriate, analysis of variance (ANOVA). The significance level was set at * *p* < 0.05 or ** *p* < 0.01.

## Supplementary Materials

Supplementary materials can be found at www.mdpi.com/1422-0067/17/12/2102/s1.

## Abbreviations

AGPAT	Acylglycerolphosphate acyltransferase
BFR	Body fat ratio
CSD	Chronic sleep deprivation
DGAT	Diacylgycerol acyltransferase
FFAs	Free fatty acids
GC-FID/MS	Gas chromatography coupled with flame ionization detector/mass spectrometry
GPR40	G-protein-coupled receptor 40
GSIS	Glucose-stimulated insulin secretion
HDL-C	High density lipoprotein-cholesterol
HFD	High fat diet
INS1	Insulin I
INS2	Insulin II
IPGTT	Intraperitoneal glucose tolerance test
IPITT	Intraperitoneal insulin tolerance test
KSIS	KCl-stimulated insulin secretion
MUFA	Monounsaturated fatty acids
ND	Normal diet
NMR	Nuclear magnetic resonance
OPLS-DA	Orthogonal projection to latent structure discriminant analysis
OGTT	Oral glucose tolerance test
PUFA	polyunsaturated fatty acids
qRT-PCR	Quantitative real-time PCR
RER	Respiratory exchange ratio
SCD1	Stearoyl CoA desaturease 1
SREBP1	Sterol-regulatory element binding proteins 1
SFA	Saturated fatty acids
SNARE	Soluble *N*-ethylmaleimide-sensitive factor attachment protein receptor
T-CHO	Total-cholesterol
TG	Triglyceride
ToFA	Total Fatty Acids
UFA	Unsaturated fatty acids
