# Supplementary material for: Decrease in Circulating Fatty Acids Is Associated with Islet Dysfunction in Chronically Sleep-Restricted Rats
Source: Int J Mol Sci. 2016 Dec 14;17(12):2102. doi: 10.3390/ijms17122102 (PMC5187902; doi:10.3390/ijms17122102)
Supplement: Supplementary file 1 [file ijms-17-02102-s001.pdf]

Supplementary Materials: Decrease in Circulating Fatty Acids is Associated with Islet Dysfunction in Chronically Sleep-Restricted Rats

Shanshan Zhan, Yangyang Wu, Peng Sun, Haiyan Lin, Yunxia Zhu and Xiao Han

Materials and Methods

^{1}H NMR Spectroscopy and Fatty Acid Analysis

Serum sample was mixed with phosphate buffer and supernatant was transferred into a NMR tube after centrifugation. NMR spectra were detected on a Bruker NMR spectrometer. OPLS-DA models were constructed with the NMR data using SIMCA-P+ (V11.0 and 12.0, Umetrics AB, Sweden). All OPLS-DA models were validated by CV-ANOVA, and $p < 0.05$ was regarded as valid. A coefficient plot was made by MatLab software. In order to analysis the composition of fatty acids, serum samples were homogenized with methanol. Internal standards, BHT and precooled acetyl chloride were added into samples. The mixture was kept at 25 °C in the dark for 24 h and then neutralized with K_2CO_3 buffer. The samples was extracted with hexane, and the supernatants were evaporated to dryness. Finally, samples were dissolved in hexane for GC−FID/MS analysis.

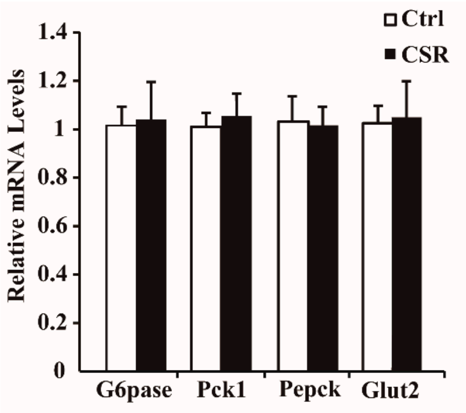

Figure S1. Chronic sleep restriction did not change mRNA expression of glucose metabolic genes in the liver of control and CSR rats. White squares = control group; black squares = CSR group. *n* = 5.

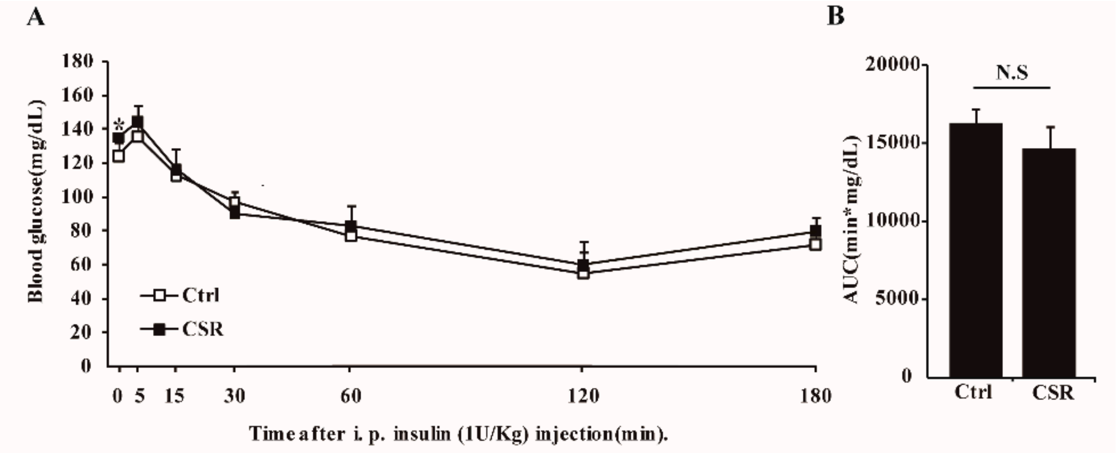

Figure S2. Chronic sleep restriction induced no changes in IPITT: (**A**) IPITTs were performed at the end of chronic sleep restriction treatment; (**B**) The AUC of (**A**). White squares = control group; black squares = CSR group. * $p < 0.05$ vs. control. N.S = no significant change. *n* = 5.

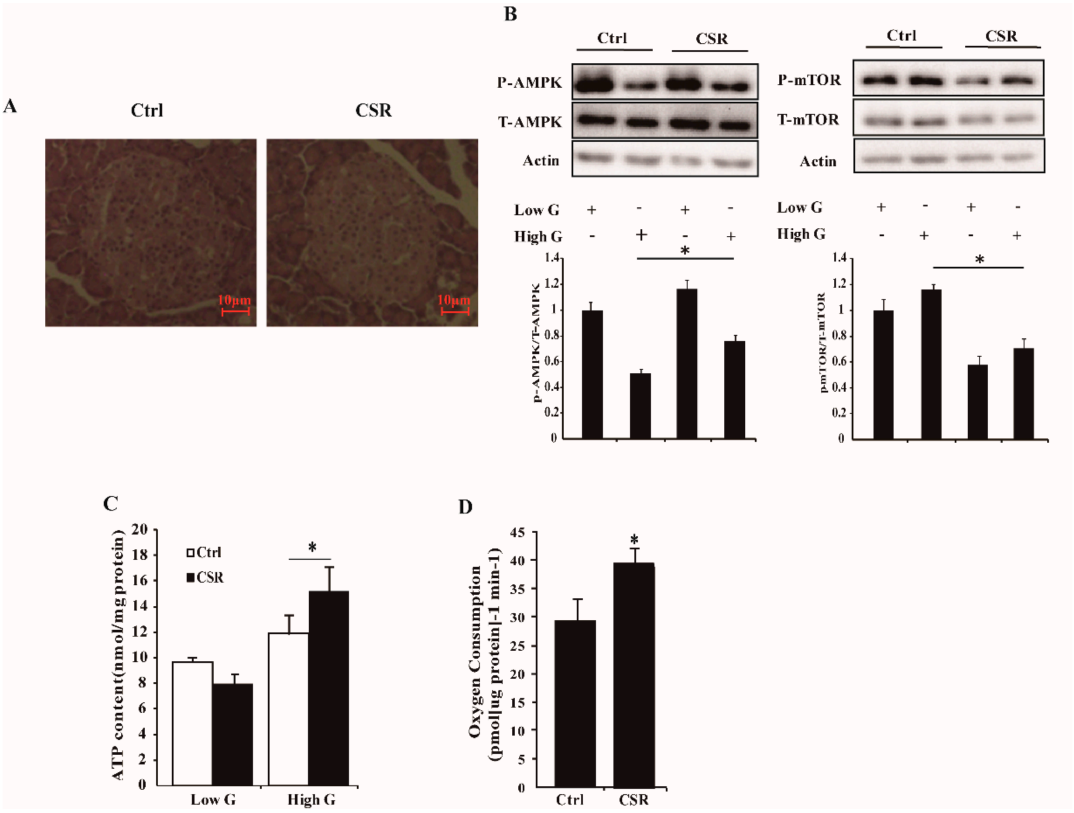

Figure S3. Chronic sleep restriction increased energy metabolism in islets of rat: (**A**) Representative morphology of islets from control and CSR rats analyzed by H&E staining; (**B**) The protein levels of AMPK and mTOR in islets treated with 3.3 mM glucose (Low **G**) and 16.7 mM glucose (High **G**); (**C**) ATP content of islets under 3.3 mM glucose (Low **G**) and 16.7 mM glucose (High **G**) was measured and normalized to total protein concentration. White squares = control group; black squares = CSR group. (D) OCR of islets under high-glucose conditions. * $p < 0.05$, vs. control. $n = 5$.

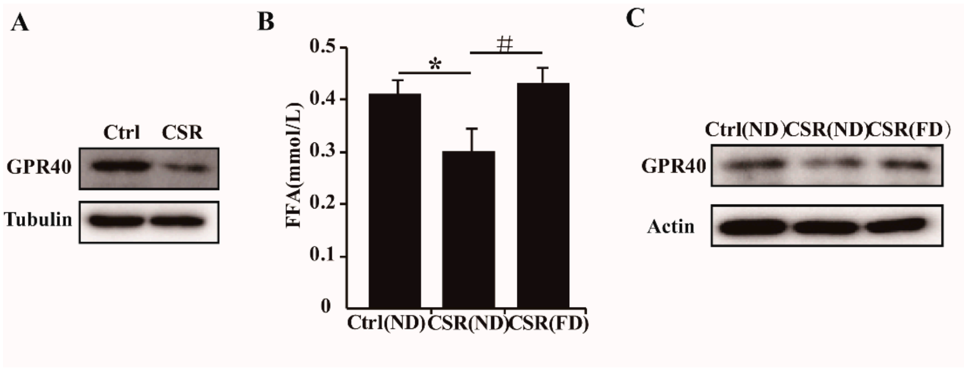

Figure S4. Chronic sleep restriction reduced rat islets response to circulating fatty acid and FD restored it partially: (**A**) Protein levels of GPR40 in isolated islets of control and CSR rats; (**B**) Concentrations of circulating free fatty acid in Ctrl(ND), CSR(ND) and CSR(FD) rats; (**C**) Protein levels of GPR40 in isolated islets of Ctrl(ND), CSR(ND) and CSR(FD) rats. ND = Normal Diet; FD = diet supplemented with fatty acids.* $p < 0.05$, Ctrl(ND) vs. CSR(ND) rats, # $p < 0.05$, CSR(FD) vs. CSR(ND) rats in (**B**). $n = 5$.

Table S1. ^{1}H NMR signal assignments of metabolites in serum.

Number	Metabolites	δ ^{1}H and Multiplicity	Proton Groups
1	Lipoproteins	0.88(m)	$-CH_3$
		1.29(m)	$-(CH_2)_n$
		1.57 (m)	$-CH_2-CH_2CO$
		2.02 (m)	$-CH_2C=C$
		2.23 (m)	$-CH_2COO$
		2.75 (b)	$-C=CCH_2C=C$
		5.31 (b)	-CH=CH-
2	Isoleucine	0.93 (t)	δ-CH_3
		1.00 (d)	γ′-CH_3
		1.28(m)	half γ-CH_2
		1.46 (m)	half γ-CH_2
		1.98 (m)	β-CH
		3.67 (dd)	α-CH
3	Leucine	0.95 (d)	δ-CH_3
		0.96 (d)	δ′-CH_3
		1.70(m)	γ-CH
		1.72 (m)	β-CH_2
		3.76 (d)	α-CH
4	Valine	0.98 (d)	γ'-CH_3
		1.04 (d)	γ-CH_3
		2.27 (m)	β-CH
		3.61 (d)	α-CH
5	3-Hydroxybutyrate	1.19 (d)	γ-CH_3
		2.31 (dd)	half α-CH
		2.41 (dd)	half α-CH
		4.17 (m)	β-CH
6	Lactate	1.32 (d)	β-CH_3
		4.11 (q)	α-CH
7	Threonine	1.32 (d)	γ-CH_3
		3.60 (d)	α-CH
		4.26 (m)	β-CH_2
8	Lysine	1.44 (m)	half δ-CH_2
		1.49 (m)	half δ-CH_2
		1.72 (m)	γ-CH_2
		1.92 (m)	β-CH_2
		3.03 (t)	ε-CH_2
		3.74 (t)	α-CH
9	Alanine	1.47 (d)	β-CH_3
		3.78 (q)	α-CH
10	Acetate	1.91 (s)	β-CH_3
11	N-acetylated glycoproteins	2.03 (s)	$-CH_3$
12	Glutamate	2.06 (m)	half β-CH_2
		2.13 (m)	half β-CH_2
		2.35 (m)	γ-CH_2
		3.75 (dd)	α-CH
13	Glutamine	2.14 (m)	β-CH_2
		2.43 (m)	γ-CH_2
		3.76 (t)	α-CH
14	O- acetylated glycoproteins	2.14 (s)	$-CH_3$
15	Deoxycytidine	1.35 (dd)	$-CH_2OH$
		6.05 (d)	5-CH(ring)
		6.27 (t)	1-CH(ribose)
		7.82 (d)	6-CH(ring)

Table S1. *Cont.*

Number	Metabolites	δ ^{1}H and Multiplicity	Proton Groups
16	Pyruvate	2.37 (s)	-C**H**$_3$
17	Citrate	2.52(d)	(a,b)half-C**H**$_2$
		2.67 (d)	(a,b)half-C**H**$_2$
18	Creatine	3.03 (s)	-C**H**$_3$
		3.93 (s)	-C**H**$_2$
19	Creatinine	3.04 (s)	-C**H**$_3$
		4.05 (s)	-C**H**$_2$
20	Tyrosine	3.06 (dd)	half β-C**H**$_2$
		3.20 (dd)	half β-C**H**$_2$
		3.94 (dd)	α-C**H**
		6.89 (d)	3,5-C**H**
		7.18 (d)	2,6-C**H**
21	Choline	3.19 (s)	-N(C**H**$_3$)$_3$
		3.53 (m)	-NC**H**$_2$
		4.10 (m)	-OC**H**$_2$
22	Phosphorylcholine	3.21 (s)	-N(C**H**$_3$)$_3$
		4.27 (m)	-OC**H**$_2$
23	β-Glucose	3.24 (m)	2-C**H**OH
		3.39 (t)	4-C**H**OH
		3.47 (m)	5-C**H**OH
		3.48 (t)	3-C**H**OH
		3.72 (m)	half-C**H**$_2$OH
		3.89 (dd)	half-C**H**$_2$OH
		4.64 (d)	1-C**H**OH
24	TMAO	3.24 (s)	-N(C**H**$_3$)$_3$
25	α-Glucose	3.40 (t)	4-C**H**OH
		3.53 (dd)	2-C**H**OH
		3.71 (t)	3-C**H**OH
		3.75 (m)	half-C**H**$_2$OH
		3.83 (m)	half-C**H**$_2$OH
		3.83 (m)	5-C**H**OH
		5.23 (d)	1-C**H**OH
26	Glycine	3.57 (s)	α-C**H**
27	Triglycerides	4.07(m)	-C**H**$_2$O
		4.28(m)	-C**H**$_2$'O
		5.21 (m)	-CHO
28	Histidine	7.05 (s)	4-C**H**
		7.78 (s)	2-C**H**
29	Phenylalanine	7.32 (dd)	2,6-C**H**
		7.37 (m)	4-C**H**
		7.42 (m)	3,5-C**H**
30	Formate	8.45 (s)	-C**H**

^{1}H NMR signal assignments of metabolites in serum. s, singlet; d, doublet; t, triplet; q, quartet; m, multiplet; b, broad peak; dd, doublet of doublets; TMAO, trimethylamine-N-oxide.

Table S2. ^1H NMR signal assignments of significantly changed metabolites in serum.

Key	δ ^1H	Moieties	Metabolites
1	0.88 (m)	$-CH_3$	Lipoproteins
	1.29 (m)	$-(CH_2)_n-$	
	1.34 (m)	$-(CH_2)_n-$	
	1.57(m)	$-CH_2CH_2CO$	
	2.02(m)	$-CH_2C=C$	
	2.23 (m)	$-CH_2COO$	
	2.75 (b)	$-C=CCH_2C=C$	
	5.31 (b)	-CH=CH-	
2	0.93 (t)	δ-CH_3	Isoleucine
	1.00 (d)	γ′-CH_3	
3	0.98 (d)	γ′-CH_3	Valine
	1.04 (d)	γ-CH_3	
4	3.19 (s)	$-N(CH_3)_3$	Choline
5	3.21 (s)	$-N(CH_3)_3$	Phosphorylcholine
6	4.07(m)	$-CH_2O$	Triglycerides
	4.28(m)	$-CH_2'O$	
	5.21 (m)	-CHO	

Table S3. Fatty acid compositions in serum.

Fatty Acids	Ctrl (μmol/L)	CSR (μmol/L)	*p* Value
C14:0	8.42 ± 1.87	6.27 ± 1.16	0.011
C16:0	523.61 ± 72.34	457.15 ± 33.55	0.026
C16:1n7	16.03 ± 4.37	11.30 ± 1.74	0.023
C18:0	131.17 ± 46.91	88.96 ± 17.02	0.031
C18:1n9	80.81 ± 14.77	63.38 ± 12.83	0.020
C18:2n6	506.60 ± 83.16	416.98 ± 67.70	0.032
C18:3n3	11.70 ± 1.49	9.99 ± 1.53	0.025
C20:0	12.78 ± 1.07	12.06 ± 1.92	0.443
C20:1	8.29 ± 0.88	7.87 ± 0.71	0.343
C20:2	5.95 ± 1.79	5.15 ± 1.22	0.336
C20:3n6	12.84 ± 4.47	9.31 ± 1.64	0.060
C20:4n6	375.66 ± 56.75	341.97 ± 23.08	0.146
C20:5n3	7.50 ± 2.63	7.94 ± 2.21	0.751
C23:0	748.11 ± 6.32	745.90 ± 4.96	0.469
C22:5	18.46 ± 6.15	13.14 ± 2.46	0.040
C22:6n3	73.39 ± 19.00	57.61 ± 10.08	0.043
ToFA	3915.14 ± 295.31	3652.09 ± 106.02	0.033
SFA	2826.73 ± 110.89	2718.54 ± 33.38	0.019
UFA	1088.41 ± 186.52	933.55 ± 78.33	0.048
PUFA	992.03 ± 171.83	838.07 ± 50.29	0.040
MUFA	102.35 ± 19.01	82.16 ± 14.30	0.031
UFA%	0.28 ± 0.03	0.25 ± 0.01	0.050
PUFA%	0.26 ± 0.02	0.23 ± 0.01	0.016
MUFA%	0.03 ± 0.003	0.02 ± 0.03	0.019
SFA%	0.76 ± 0.03	0.73 ± 0.01	0.075
PUFA/MUFA	9.55 ± 1.17	10.44 ± 1.62	0.186
PUFA/UFA	0.91 ± 0.01	0.92 ± 0.01	0.155

Data are expressed as "mean ± SD". * $p < 0.05$.ToFA: total fatty acids; UFA: unsaturated fatty acids; PUFA: polyunsaturated fatty acids; MUFA: monounsaturated fatty acids; SFA: saturated fatty acids; PUFA/MUFA: PUFA-to-MUFA ratio; PUFA/UFA: PUFA-to-UFA ratio; MUFA/UFA: MUFA-to-UFA ratio; PUFA/SFA: PUFA-to-SFA ratio;n3: n3 PUFA; n6: n6 PUFA; n6/n3: n6 PUFA-to-n3 PUFA ratio.
